# Establishing anchor-based minimal important change values for the functional independence measure in older patients with vertebral compression fractures in Japan: A retrospective observational study

**DOI:** 10.1097/MD.0000000000047936

**Published:** 2026-03-06

**Authors:** Takumi Igusa, Hiroyuki Uchida, Tomoaki Shirakawa, Kazuki Ishii, Yudai Kato, Yuki Yamajo, Chihaya Machida, Tomohiro Shimada, Kenji Tsuchiya, Senichiro Kikuchi, Kazuki Hirao

**Affiliations:** a Graduate School of Health Sciences, Gunma University, Maebashi, Japan; b Department of Rehabilitation, Kurashiki Heisei Hospital, Kurashiki, Japan; c Department of Rehabilitation, Fujioka General Hospital, Fujioka, Japan; d Department of Rehabilitation, Medical Corporation Taiseikai, Uchida Hospital, Numata, Japan; e Department of Rehabilitation, Faculty of Health Sciences, Nagano University of Health and Medicine, Nagano, Japan; f Department of Occupational Therapy, School of Health Sciences, Faculty of Medicine, Kagoshima University, Kagoshima, Japan.

**Keywords:** activities of daily living, functional independence measure, minimal important change, rehabilitation, vertebral compression fractures

## Abstract

In rehabilitation medicine, the functional independence measure (FIM) is widely used to assess activities of daily living, but its minimal important change (MIC) in patients with vertebral compression fractures (VCFs) remains unclear. The MIC is defined as the smallest within-person change perceived by patients or clinicians to represent an important improvement. The MIC is crucial in evaluating the effectiveness of interventions across various studies and in setting of treatment goals. Accordingly, this study aimed to estimate the MIC of the FIM in patients with VCFs. This retrospective observational study estimated the MIC (the receiver operating characteristic method [minimal important change estimated using the receiver operating characteristic method [MIC_roc_]], the predictive modeling method [minimal important change estimated using the predictive modeling method [MIC_pred_]], the adjusted predictive modeling method [adjusted minimal important change [MIC_adj_]]) of the FIM in 290 older patients with VCFs admitted at a convalescent rehabilitation ward in Japan between January 2020 and December 2022, using discharge destination (home vs elsewhere) as the anchor. The sample size was determined in accordance with the recommended criterion for MIC estimation (≥100 participants), and all eligible participants available during the study period were included. The 95% confidence intervals for the MIC estimated using each method (MIC_roc_, MIC_pred_, and MIC_adj_) were calculated using the bootstrap method (n = 2000). The mean age of the participants was 82.61 ± 7.08 years, and 216 (74.48%) were female. While 254 participants (87.59%) were discharged home, 36 (12.41%) were discharged to other facilities, such as nursing homes. The MIC_roc_, MIC_pred_, and MIC_adj_, respectively, were 31.5, 26.39, and 22.15 for functional independence measure motor items, while these were 0.5, 0.6, and −1.09 for functional independence measure cognitive items. For functional independence measure total score, these were 40.5, 27.01, and 21.53, respectively. This study is the first to estimate the MIC of the FIM using multiple methods in older patients with VCFs admitted at a convalescent rehabilitation ward, providing new insights into the definition of clinically meaningful changes in rehabilitation assessment. Future studies should incorporate multidimensional anchors, including patient-reported outcomes, and employ more rigorous prospective study designs and multicenter collaborations.

## 1. Introduction

Vertebral compression fractures (VCFs) predominantly occur in older adults, often among those with underlying conditions such as osteoporosis.^[1,2]^ In such cases, the vertebral body of the spine partially or completely collapses, resulting in spinal deformity, postural changes, pain, and reduced range of motion.^[3–5]^ Consequently, basic movements such as standing and walking are often impaired, greatly impacting patients’ independence in activities of daily living (ADLs) and quality of life.^[5–10]^ In patients with VCFs, the short-term goal is to alleviate symptoms and enable early mobilization, while the long-term goal is to improve mobility and function, ultimately to regain independence in ADLs.^[11]^

In recent years, various rehabilitation programs aimed at improving ADLs and enhancing activity have been developed for patients with VCFs.^[12–14]^ These programs must be evaluated through assessment scales that demonstrate high reliability and validity, as well as strong sensitivity. Several scales are used to assess function in these patients,^[9,15–18]^ including the functional independence measure (FIM),^[19–21]^ which is used to assess ADLs in patients with VCFs worldwide. The FIM covers a range of ADL items such as self-care, transfers, mobility, and toileting, and it is widely used as an index for comprehensively assessing independence.^[22,23]^ The FIM includes 13 motor items and 5 cognitive items, which are each scored from 1 to 7 for a total score ranging from 18 to 126, with higher scores indicating less need for assistance and fewer activity limitations. Many studies have evaluated the reliability and validity of the FIM across various diseases.^[19,20,24–28]^ Since changes in FIM scores enable an objective evaluation of patients’ functional recovery, it is widely used in both clinical practice and research.^[19–21]^ However, to truly confirm its utility, it is also crucial to determine the interpretability of the FIM, specifically the minimal important change (MIC).

The MIC is defined as the smallest within-person change perceived by patients or clinicians to represent an important improvement. In other words, any change exceeding the MIC is interpreted as clinically meaningful.^[29]^ The MIC is crucial in evaluating the effectiveness of interventions across various studies and in setting of treatment goals.^[29]^ Clinical research and practice should emphasize not only statistical data, but also clinical differences, integrating both key aspects together.^[30,31]^ Thus, estimating the MIC of the FIM will be highly valuable in future clinical research and clinical practice. To date, many studies have estimated the MIC of the FIM for stroke patients and patients with femoral neck fractures, but not in patients with VCFs.^[32,33]^

Understanding the MIC of the FIM enables a more clinically relevant evaluation of rehabilitation interventions, which can provide meaningful insights for developing more effective treatment strategies to improve ADLs in patients with VCFs. Accordingly, this study aimed to estimate the MIC of the FIM in patients with VCFs.

## 2. Methods

### 2.1. Study design and participants

This retrospective observational study evaluated older patients with VCFs admitted at the convalescent rehabilitation ward (CRW) of Kurashiki Heisei Hospital in Okayama Prefecture, Japan between January 1, 2020, and December 31, 2022. In this retrospective observational study, purposive sampling was applied. All patients who met the predefined inclusion criteria during the study period were retrospectively identified using electronic medical records. The inclusion criteria were as follows: males or females aged ≥65 years, diagnosed with VCFs, native Japanese speakers, and living at home prior to admission. The exclusion criteria were as follows: death during hospitalization, and transfer to an acute care ward due to deteriorating clinical status. For patients hospitalized multiple times during the study period, only the first admission was included in the analysis.

Written informed consent was waived because this was a retrospective observational study using existing medical information obtained during routine clinical care. Instead, a summary of the study was disclosed on the official website of Kurashiki Heisei Hospital, and participants were given the opportunity to refuse the use of their data (opt-out). This study was approved by The Ethical Review Board for Medical Research Involving Human Subjects of Gunma University (Approval No.: HS2024-067).

### 2.2. Measurements

#### 2.2.1. Patient demographics and clinical characteristics

The following data was collected: sex, age, height, weight, body mass index, type of VCFs, FIM at admission, FIM at discharge, length of hospital stay, and discharge destination. VCFs were classified with reference to previous studies.^[13,34,35]^

#### 2.2.2. Functional independence measure

The FIM is a scale used to assess the degree of activity limitation by observing participants’ behavior and measuring the degree of assistance needed to perform basic physical and cognitive activities.^[22,23]^ The scale measures 18 items: 13 motor items (functional independence measure motor items [FIM_motor_]: eating, grooming, bathing, dressing the upper body, dressing the lower body, toileting, bladder management, bowel management, transfer to bed/chair/wheelchair, transfer to toilet, transfer tub/shower, walk or wheelchair, and stairs) and 5 cognitive items (functional independence measure cognitive items [FIM_cognitive_]: comprehension, expression, social interaction, problem solving, and memory). Each item is rated on an ordinal scale from 1 (i.e., total assistance) to 7 (i.e., complete independence), with a total score (functional independence measure total score [FIM_total_]) ranging from 18 to 126 points (FIM_motor_: 13–91 points, FIM_cognitive_: 5–35 points). Higher scores indicate greater independence and less need for assistance. Trained therapists conducted the FIM assessments, both upon admission and at discharge.

### 2.3. Sample size

It has been suggested that a minimum of 100 patients is required to estimate the MIC.^[29]^ Therefore, we determined that collecting data from January 1, 2020, to December 31, 2022, would enable achievement of the target sample size (i.e., ≥100 patients).

### 2.4. Statistical analysis

#### 2.4.1. Minimal important change

Methods for estimating the MIC can be broadly divided into 2 categories: distribution-based methods and anchor-based methods. Anchor-based methods, which include the mean change method, the receiver operator characteristic method (ROC) method, predictive modeling method, and vignette-based method, are generally considered more appropriate for estimating the MIC.^[29]^ Notably, the mean change and vignette-based methods have certain limitations, with possible issues regarding estimation accuracy.^[29]^ However, the ROC and predictive modeling methods are generally considered as the more appropriate anchor-based approaches for estimating the MIC. The ROC method is the most commonly used approach, but it is highly sensitive to random sampling variability and cannot fully account for external factors that may influence the MIC.^[29,36]^ Additionally, bias can occur when the improvement rate of the dichotomized anchor deviates from 50%, possibly over- or underestimating the true MIC.^[29,36]^ In contrast, the predictive modeling method addresses these limitations of the ROC method (i.e., random sampling variability and external factors influencing the MIC),^[29,36]^ as well as the bias that arises when the improvement rate of the dichotomized anchor deviates from 50%.^[29,36]^

#### 2.4.2. External anchors for MIC validation

The outcome anchor used in this study was the discharge destination from the CRW (home vs other), which was set as a binary variable. In general, when estimating the MIC using an anchor-based approach, self-administered questionnaires with a single item are often employed (e.g., a 5- or 7-point scale ranging from “much worse” to “much improved”).^[29]^ However, it was not feasible to use a self-administered questionnaire as the anchor of this study due to its retrospective cohort design. Therefore, considering that the CRW aims to support patients in returning home, discharge destination from the CRW was used as the anchor. Similarly, previous studies have also used the discharge destination (home vs elsewhere) as an anchor to estimate the MIC of the Barthel Index, an assessment scale for ADL.^[37]^ Since older adults often strongly desire to be discharged home and live independently in their own residences, using the discharge destination as an anchor is appropriate.^[38–42]^

#### 2.4.3. Methods for calculating MIC

All analyses were performed using R (version 4.5.0; R Foundation for Statistical Computing, Vienna, Austria). MIC was estimated using the minimal important change estimated using the receiver operating characteristic method (MIC_roc_) and the predictive modeling method (minimal important change estimated using the predictive modeling method [MIC_pred_]), among which the latter is generally more accurate.^[29,36]^ MIC_pred_ was calculated using the following formula:


$$\begin{array}{l} MIC_{pred}=\frac{In\left(odds_{pre}\right)-C}{B} \end{array}$$

In this formula, *C* represents the intercept in the logistic regression analysis, while *B* denotes the regression coefficient. In the logistic regression analysis, the dependent variable was the dichotomized anchor (discharge destination from the CRW: home vs elsewhere), and the independent variable was the change in FIM score from admission to discharge.^[29,36]^ In addition, odds_pre_ was calculated based on the improvement rate of the anchor using the following formula:


$$\begin{array}{l} odds_{pre}=\frac{Prevalence}{1-prevalence} \end{array}$$

In the ROC method, bias is likely when the improvement rate of the anchor is not 50%. In contrast, the predictive modeling method incorporates a correction formula that is specifically designed to account for this bias. Shown below is the formula for the adjusted minimal important change (MIC_adj_), which is derived from the predictive modeling method.^[29,36]^ In this formula, Cor represents the correlation coefficient between the dichotomized anchor and change in FIM, while SD_change_ denotes the standard deviation of the change in FIM.


$$\begin{array}{l} MIC_{adj}=MIC_{pred}-S\times In\;\left(odds_{pre}\right) \end{array}$$

$$\begin{array}{l} S=0.09\times SD_{change}+0.103\times SD_{change}\times Cor \end{array}$$

The 95% confidence intervals for the MIC estimated using each method (MIC_roc_, MIC_pred_, and MIC_adj_) were calculated using the bootstrap method (n = 2000).^[29,36]^

### 2.5. Ceiling effect and floor effect

Floor and ceiling effects indicate the proportion of respondents who obtain the lowest (floor) or highest (ceiling) possible scores; these serve as important indicators for evaluating the sensitivity, score range, and discriminative ability of an assessment scale.^[43]^ High floor or ceiling effects can compromise the reliability and validity of measurements, which can influence the estimation of MIC.^[43–46]^ In previous studies, floor or ceiling effects were considered to be present when >15% of respondents achieved the minimum or maximum score, respectively, and this was also adopted for the present study.^[47,48]^ Specifically, a floor effect was deemed present if >15% of patients demonstrated the lowest score at baseline (motor items: 13 points, cognitive items: 5 points, total FIM: 18 points), while a ceiling effect was deemed present when >15% of patients achieved the highest score (motor items: 91 points, cognitive items: 35 points, total FIM: 126 points).^[47,48]^

## 3. Results

### 3.1. Characteristics of the participants

The eligibility criteria was fulfilled by 290 out of 1401 patients admitted to the CRW between 2020 and 2022, and their demographic and baseline clinical characteristics are presented in Table 1. The mean age of the participants was 82.61 ± 7.08 years, and 216 (74.48%) were female. While 254 participants (87.59%) were discharged home, 36 (12.41%) were discharged to other facilities, such as nursing homes. The mean FIM_motor_, FIM_cognitive_, and FIM_total_ scores, respectively, were 40.27 ± 14.66, 26.31 ± 7.06, and 66.58 ± 18.87 points upon admission, whereas these were 68.88 ± 16.50, 27.37 ± 6.83, and 96.25 ± 22.13 points at discharge. The changes in FIM scores from admission to discharge were 28.61 ± 21.86 for FIM_motor_, 1.06 ± 9.21 for FIM_cognitive_, and 29.67 ± 28.45 for FIM_total_.

**Table 1:** Characteristics of the participants (n = 290).

Characteristics	N = 290
Age (yr)	82.61 (7.08)
Sex	
Female	216 (74.48%)
Male	74 (25.52%)
Height	152.92 (7.49)
Weight	50.59 (9.76)
BMI	21.60 (3.69)
Type of fracture	
Lumbar vertebra	165 (41.38%)
Thoracic vertebra	120 (56.9%)
Both thoracic and lumbar vertebra	5 (1.72%)
Length of stay (days)	56.74 (23.77)
Discharge destination	
Home	254 (87.59%)
Other	36 (12.41%)
FIM at admission	
Motor	40.27 (14.66)
Cognitive	26.31 (7.06)
Total	66.58 (18.87)
FIM at discharge	
Motor	68.88 (16.50)
Cognitive	27.37 (6.83)
Total	96.25 (22.13)
Change FIM	
Motor	28.61 (21.86)
Cognitive	1.06 (9.21)
Total	29.67 (28.45)
Data are means (standard deviation) or numbers (%)	

BMI = body mass index, FIM = functional independence measure.

### 3.2. Minimal important change

Table 2 presents the values of MIC_roc_, MIC_pred_, and MIC_adj_ for the FIM. For FIM_motor_, FIM_cognitive_, and FIM_total_, respectively, the estimated MIC_roc_ values were 31.5 points (95% CI, −12.00 to 42.50), 0.5 points (95% CI, −8.50 to 9.50), and 40.5 points (95% CI, −12.50 to 57.43), whereas the estimated MIC_pred_ values were 26.39 points (95% CI, 22.27–30.40), 0.6 points (95% CI, −1.04 to 2.23), and 27.01 points (95% CI, 21.71–32.26). Meanwhile, the estimated MIC_adj_ values were 22.15 points (95% CI, 17.29–26.79), −1.09 points (95% CI, −2.98 to 0.70), and 21.53 points (95% CI, 15.21–27.37), respectively. The correlation coefficients between the dichotomized anchor and the changes in FIM scores were 0.091, 0.043, and 0.084 for FIM_motor_, FIM_cognitive_, and FIM_total_, respectively.

**Table 2:** MIC estimation with the ROC and predictive modeling methods (n = 290).

	MIC_roc_	95% CI	MIC_pre_	95% CI	MIC_adj_	95% CI
FIM_motor_	31.5	−12.00 to 42.50	26.39	22.27 to 30.40	22.15	17.29 to 26.79
FIM_cognitive_	0.5	−8.50 to 9.50	0.6	−1.04 to 2.23	−1.09	−2.98 to 0.70
FIM_total_	40.5	−12.50 to 57.43	27.01	21.71 to 32.26	21.53	15.21 to 27.37

FIM = functional independence measure, FIM_cognitive_ = functional independence measure cognitive items, FIM_motor_ = functional independence measure motor items, FIM_total_ = functional independence measure total score, MIC = minimal important change, MIC_adj_ = adjusted minimal important change, MIC_pred_ = minimal important change estimated using the predictive modeling method, MIC_roc_ = minimal important change estimated using the receiver operating characteristic method.

### 3.3. Floor and ceiling effects

No floor or ceiling effects were observed for FIM_motor_, FIM_cognitive_, or FIM_total_.

## 4. Discussion

This study is the first to estimate the MIC of the FIM using multiple methods among older patients with VCFs admitted at a CRW. Enhancing functional independence is essential for maintaining quality of life among patients with VCFs, and the findings of this study are valuable in contributing toward this goal.

The MIC_roc_, MIC_pred_, and MIC_adj_, respectively, were 31.5 (95% CI, −12.00 to 42.50), 26.39 (95% CI, 22.27–30.40), and 22.15 (95% CI, 17.29–26.79) for FIM_motor_, while these were 0.5 (95% CI, −8.50 to 9.50), 0.6 (95% CI, −1.04 to 2.23), and −1.09 (95% CI, −2.98 to 0.70) for FIM_cognitive_. For FIM_total_, these were 40.5 (95% CI, −12.50 to 57.43), 27.01 (95% CI, 21.71–32.26), and 21.53 (95% CI, 15.21–27.37), respectively. These findings indicate that the estimated values of MIC vary depending on the estimation method, which is consistent with previous research.^[49–51]^ However, the MIC_roc_ may have been inflated due to imbalances in the improvement rate of the anchor and also because around 88% of the participants were classified as improved (i.e., discharged home).^[29]^ Furthermore, the MIC_roc_ had wide confidence intervals, with some including zero, suggesting uncertainty in the estimation. Thus, greater emphasis should be placed on the MIC_adj_, which accounts for the imbalance in the anchor ratio, rather than relying solely on the values derived from the ROC method. Since FIM scores are assessed as integer values, the MIC should be 23 points for FIM_motor_ and 22 points for FIM_total_.

Regarding FIM_cognitive_, although an MIC_adj_ value was calculated, its value fell below zero and therefore could not be utilized as a valid MIC for the FIM. This negative value was likely obtained because of the effect of the adjustment resulting from the imbalance in the proportion of patients classified as improved.^[29,52]^ MIC_adj_ is calculated by applying an adjustment to MIC_pred_ that accounts for the proportion of patients classified as improved. Specifically, when the proportion of patients that improved markedly deviates from 50%, the adjustment term increases, causing MIC_adj_ to be smaller than MIC_pred_.^[29,52]^ As a result, MIC_adj_ may fall below zero. However, this does not indicate anomalies in the data but, rather, represents a theoretically explicable phenomenon arising from the process of correcting the bias inherent to MIC_pred_. Additionally, the study population already had relatively high cognitive scores upon admission, resulting in limited room for change. In fact, the mean FIM_cognitive_ scores were 26.31 ± 7.06 upon admission and 27.37 ± 6.83 at discharge, resulting in a minimal change of only 1.06 ± 9.21. Likewise, previous studies have also noted that the discriminative ability of the MIC decreases in populations with high scores upon admission.^[32]^

The findings of this study are novel and clinically valuable, potentially serving as a reference for subsequent research on the MIC of the FIM. The MIC is valuable not only in clinical practice but also for determining the effects of interventions in clinical research.^[29]^ However, several aspects of the calculated MIC warrant cautious interpretation. When estimating the MIC, the validity of the chosen anchor is highly important, and a correlation of at least 0.3 between the scale and the anchor is considered desirable.^[29]^ In the present study, the correlation coefficients between the change in FIM and the anchor were 0.091 for FIM_motor_ and 0.084 for FIM_total_, indicating that the anchor (i.e., discharge destination) may not have functioned adequately. This could be attributed to the influence of the unique public long-term care insurance (LTCI) system in Japan.^[53,54]^ Under the LTCI system, older adults aged 65 years and above are eligible to receive LTCI services either at home or in facilities, regardless of income level or the availability of family caregiving, with 5.75 million out of 35.79 million older adults utilizing these services in 2020.^[53,54]^ Therefore, even if patients have substantial ADL limitations, support from the LTCI system (e.g., home-visit care and housing modifications) often enables discharge to home, which could contribute to the weak correlation between changes in FIM scores and discharge destination. Thus, the context of this specific study population and the healthcare system in Japan may have had a considerable influence on our findings. Nevertheless, selecting more valid anchors remains a challenge for future research. Using multiple anchors, including patient-reported outcomes, can further improve the accuracy of MIC estimation.

This study has several limitations. First, due to the retrospective study design, the data was not specifically collected for the study objectives, making it impossible to completely eliminate sampling bias or missing information. Second, objective indicators were used as anchors to determine the MIC, without incorporating patient-reported outcome measures. Consequently, the MIC of the FIM estimated in this study may not fully reflect patients’ subjective evaluations. Third, because this study used data from a single medical institution in Japan, caution is required when applying these findings across patient populations from other facilities or regions. Future research should implement a prospective cohort study design, incorporate patient-reported outcomes into the anchors, and accumulate varied data from multicenter collaborative studies. Fourth, the MIC was not estimated using stratification by age, sex, fracture site, or comorbidities. At the study design stage, it was anticipated that obtaining a sufficient sample size to permit stratified analyses (i.e., at least 100 participants per group) would be challenging. Moreover, because prior studies have generally not estimated MICs using stratified approaches, stratification was not performed in the present study. However, it is possible that the estimated MIC varies according to factors such as age or sex. In particular, because women constituted a large proportion of the study population, the generalizability of the MIC derived from this study to male patients may be limited. Future research should estimate MICs while accounting for variables such as age, sex, fracture site, and comorbidities. Nevertheless, our findings provide a new foundation for estimating the MIC of the FIM in older patients with VCFs, which can serve as a useful indicator in future rehabilitation research and clinical practice.

## 5. Conclusion

This study is the first to estimate the MIC of the FIM using multiple methods in older patients with VCFs admitted at a CRW, providing new insights into the definition of clinically meaningful changes in rehabilitation assessment. The estimated MIC values varied depending on the method used, with the ROC method being particular susceptible to the effects of bias in the proportion of improved patients and uncertainty in estimation. The adjusted predictive modeling method offered the best estimation of the MIC, which was 23 points for FIM_motor_ and 22 points for FIM_total_. However, the estimated MIC for FIM_cognitive_ was below zero, which is not feasible for use. Moreover, the anchor used in this study (i.e., discharge destination) had limited validity due to its low correlation with FIM scores, likely influenced by the LTCI system in Japan. Future studies should incorporate multidimensional anchors, including patient-reported outcomes, and employ more rigorous prospective study designs and multicenter collaborations. Nevertheless, these findings provide an important starting point for estimating the MIC of FIM in patients with VCFs, which can be valuable in both future research and clinical practice.

## Acknowledgments

This work was supported by the rehabilitation staff at Kurashiki Heisei Hospital for their invaluable cooperation in this study.

## Author contributions

**Conceptualization:** Takumi Igusa, Hiroyuki Uchida, Kazuki Hirao.

**Data curation:** Takumi Igusa, Hiroyuki Uchida.

**Formal analysis:** Takumi Igusa, Hiroyuki Uchida.

**Investigation:** Hiroyuki Uchida, Tomoaki Shirakawa, Kazuki Ishii, Yudai Kato, Yuki Yamajo.

**Methodology:** Takumi Igusa, Hiroyuki Uchida, Kazuki Hirao.

**Project administration:** Takumi Igusa.

**Resources:** Hiroyuki Uchida, Kazuki Hirao.

**Supervision:** Kazuki Hirao.

**Writing – original draft:** Takumi Igusa, Hiroyuki Uchida, Kazuki Hirao.

**Writing – review & editing:** Takumi Igusa, Hiroyuki Uchida, Tomoaki Shirakawa, Kazuki Ishii, Yudai Kato, Yuki Yamajo, Chihaya Machida, Tomohiro Shimada, Kenji Tsuchiya, Senichiro Kikuchi, Kazuki Hirao.
